# Addressing budget reduction and reallocation on health-related resources during COVID-19 pandemic in malaria-endemic countries

**DOI:** 10.1186/s12936-020-03488-y

**Published:** 2020-11-16

**Authors:** Ajib Diptyanusa, Karen Nelwin Zablon

**Affiliations:** 1 Department of Parasitology, Faculty of Medicine, Public Health and Nursing, Universitas Gadjah Mada, Jalan Farmako, Sekip Utara, Yogyakarta, 55281 Indonesia; 2 National Institute for Medical Research, Mwanza, Tanzania

**Keywords:** Malaria, Budget, Reduction, Reallocation, Funding, Resources, COVID

## Abstract

The global COVID-19 pandemic has been affecting the maintenance of various disease control programmes, including malaria. In some malaria-endemic countries, funding and personnel reallocations were executed from malaria control programmes to support COVID-19 response efforts, resulting mainly in interruptions of disease control activities and reduced capabilities of health system. While it is principal to drive national budget rearrangements during the pandemic, the long-standing malaria control programmes should not be left behind in order to sustain the achievements from the previous years. With different levels of intensity, many countries have been struggling to improve the health system resilience and to mitigate the unavoidable stagnation of malaria control programmes. Current opinion emphasized the impacts of budget reprioritization on malaria-related resources during COVID-19 pandemic in malaria endemic countries in Africa and Southeast Asia, and feasible attempts that can be taken to lessen these impacts.

## Background

Great achievements in reducing the global malaria burden have been accomplished over the past decades, with 11 countries achieving malaria elimination. Such accomplishments have been made possible largely to successful prevention and control programme, including the distribution of insecticide-treated bed nets (ITNs) and long-lasting insecticide-treated bed nets (LLINs), indoor residual spraying (IRS), and more effective antimalarial treatments [1]. However, the ongoing pandemic of corona virus disease of 2019 (COVID-19) has caused disruptions to health services in malaria elimination through various ways: through overwhelmed health system in response to the pandemic, through the delay in the operations of malaria control programmes, and through interrupted supplies of anti-malarial drugs. The disruptions in services for malaria in low- and middle-income endemic countries could lead to additional loss of life. When the disruptions of the malaria control programme persists, it had been estimated that malaria-related deaths over 5 years may be increased by up to 36% in high burden settings, compared to if there were no COVID-19 epidemic [2].

The rapid spreading of COVID-19 stated as a pandemic in the first quarter of 2020 pushes immediate actions from multidisciplinary stakeholders to reduce the morbidity and mortality of the disease. The actions taken from the authorities might and already has had an impact on funding allocation on other aspects, particularly in infrastructure development, popular term called as the infrastructure gap. In developing countries, this particular issue might have surfaced the earliest. In some countries, however, funding might have been cut down mainly from public health sector unlike other sectors to aid for COVID-19 management and containment, particularly from other disease control programmes, including malaria. The continuation of malaria control programme is essential in tropical areas in Africa and Southeast Asia, where the highest burden of malaria occur, otherwise substantial progress in malaria elimination will be put into jeopardy hence threatening the targets of global malaria elimination and eradication strategy. Current opinion focuses on the inevitable impacts of budget reduction and reallocation on malaria-related resources during COVID-19 pandemic in high malaria endemic countries in Africa and Southeast Asia, and possible attempts that can be taken to mitigate these impacts.

## Main text

Malaria control is one of the longest running health programmes at the Ministry of Health (MoH) in malaria endemic countries, including in Indonesia, Thailand and Cambodia in Southeast Asia, and in Tanzania, Mali and Ghana in Africa [3, 4]. In these countries, the programmes run in malaria control are usually carried out by the teams in the National Malaria Control Programme (NMCP) that commonly belong to the MoH of their respective countries [5]. The current main strategies in malaria control programme include enhanced laboratory diagnosis, early treatment of malaria cases with anti-malarial drugs and interventions to reduce human-mosquito contact, such as IRS, larviciding, distribution of ITNs or LLINs, with additional programmes that may differ in several countries [6]. It has been emphasized by the World Health Organization (WHO) that while protecting health workers as well as communities against COVID-19 transmission, maintenance of malaria control programmes in malaria-affected countries should remain vital. In fact, several countries in sub-Saharan Africa, namely Côte d'Ivoire, Comoros, and Ghana, had deferred the ITNs and IRS campaigns during the COVID-19 pandemic when such campaigns have been the mainstay of malaria control efforts in the region for decades [7, 8]. A modelling analysis by the WHO estimated a doubling in the number of malaria mortality rate in 2020 if ITN campaigns were suspended and access to anti-malarial drugs were reduced [8]. As the impact of the COVID-19 pandemic on the established malaria control programme in endemic countries has been acknowledged as substantial, priorities should be made to address the threats COVID-19 poses to the malaria control programme. The estimated greatest threat in malaria control programme during current COVID-19 pandemic is the reduced prevention activities [2]. These include scaling back of control activities, reduced capabilities of the overwhelmed health system, and substandard production of anti-malarial drugs driven by cost cutting. However, resources including funds and personnel are being reallocated from malaria and other programmes to support COVID-19 response efforts [9].

Further delay in infrastructure development in current pandemic era has taken its toll on the interruption of medical supplies distribution in low-income countries both in Africa and Southeast Asia regions, making it more challenging to accommodate test kits and ITNs in remote areas. Presence of COVID-19 has greatly influenced the delay of continuation of the ongoing IRS and larviciding activities in Tanzania and Uganda, resulting to minimal coverage of the targeted houses and mosquito breeding hubs [10]. Inevitable reduction of malaria targeted sites due to budget reduction and reallocation have led to unstable tracking of malaria prevalence post IRS hindrance in these areas [11]. While in some of the countries in Southeast Asia, including Indonesia, almost all malaria activities planned for 2020, such as active case finding through house-to-house visits by community health workers, distribution of LLINs, and migration surveillance have been disrupted due to reprioritization of the state budget and circuit breaker (lockdown) policy in response to COVID-19 pandemic. This has resulted in over 50% decline in active and passive case finding of malaria, where only about 500,000 suspected cases got tested from January through May 2020 alone [12]. Additionally, insufficient personal protective equipment and growing stigmatization over potential source of COVID-19 transmission source further affect these malaria workers [9]. Tailoring malaria intervention strategies in the COVID-19 response has been suggested by the WHO to ensure service deliveries and to prevent fatal consequences of malaria.

In response to previous success stories in COVID-19 clinical case management in various parts of the world, the anti-malarial drugs chloroquine, hydroxychloroquine and artemisinin-based therapy have been widely used to treat the patients diagnosed with COVID-19 in addition to standard supportive care [13, 14]. Reports of bulk procurement from foreign pharmaceutical companies and third-party wholesale distributors by the government or people in panic, have resulted in global shortage of anti-malarial drugs [15]. Consequently, the practice of urgent policies has been implemented in several Southeast Asian countries in order to sustain the access to these drugs. These include local production of drugs similar to those available in the market, substituting the major drug component chloroquine phosphate with quinine sulfate; these drugs certainly need efficacy evaluation. This production of substandard drugs was also driven by cost cutting and supply chain errors [16]. Nevertheless, approximately 95% of the budget that was cut from the Indonesian Department of Research and Development had made it almost impossible to perform trials using this potential drug for COVID-19.

To minimize the impact of COVID-19 disruptions, continuous supply of diagnostic kits and adequate support of ITN, IRS and other preventive actions are utmost important. Maintenance of anti-malarial drug quality is also vital. Particularly in developing countries in Africa and Southeast Asia, these aspects are believed to be better supported by additional funding sources, as tremendous economic and social damage caused by the pandemic could be sustained longer than developed countries. However, additional funding supports from private sectors, international organizations, monetary cooperation and developed countries less-affected by COVID-19 were apparently missing their targets by not directing the aids in these particular regions which is explainable due to the fact that the world’s economy itself has dropped due to the unexpected pandemic. It might also be too much to expect the establishment of a global fund for health during this era [17]. Nevertheless, whilst these countries are currently fighting their respective battles against COVID-19 with differing levels of intensities, exercising options in reprioritization of state budget without creating a crisis for the handling of fatal diseases such as malaria during the pandemic should be encouraged. Lessons learned from the current global COVID-19 pandemic should be able to improve the health system resilience in developing countries in Africa and Southeast Asia, mainly the capacity of health institutions and populations to prepare for and effectively respond to crises [18].

## Conclusions

The ongoing COVID-19 pandemic has greatly affected the system of governance in several health sectors, and that malaria-affected countries in African and Southeast Asian regions must use the current opportunity to harvest new approaches for the future. Immediate support is required to review the achievements towards indicators of certification of malaria elimination, principally the mitigation of negative impacts of COVID-19 pandemic, during which malaria elimination stagnation is seemingly inevitable. Lastly, to sustain malaria control services in COVID-19 settings, the following qualities are urgently required: countries who cooperate, governments who deliver, and communities who mobilize.

## Data Availability

Data sharing is not applicable to this article as no datasets were generated or analysed during the current study.

## Abbreviations

COVID-19: Coronavirus disease of 2019
IRS: Indoor residual spraying
ITN: Insecticide-treated net
LLIN: Long-lasting insecticide-treated bed net
MoH: Ministry of Health
NMCP: National Malaria Control Programme
WHO: World Health Organization
